# Understanding Anxiety Symptoms of Mood Disorders Across Bipolar and Major Depressive Disorder Using Network Analysis

**DOI:** 10.3390/medicina61122245

**Published:** 2025-12-18

**Authors:** Sarah Soonji Kwon, Hyukjun Lee, Jakyung Lee, Junwoo Jang, Daseul Lee, Hyeona Yu, Hyo Shin Kang, Tae Hyon Ha, Jungkyu Park, Woojae Myung

**Affiliations:** 1 Department of Psychiatry, Huntsman Mental Health Institute, University of Utah, Salt Lake City, UT 84108, USA; sarah.kwon@hsc.utah.edu; 2 Department of Neuropsychiatry, Seoul National University Bundang Hospital, Seongnam 13620, Republic of Korea; lhj4071@gmail.com (H.L.); jklee96@ewhain.net (J.L.); julian6312@gmail.com (J.J.); daseul6866@naver.com (D.L.); hkhkh2142@naver.com (H.Y.); hatti@snu.ac.kr (T.H.H.); 3 Department of Psychology, Kyungpook National University, Daegu 41566, Republic of Korea; hyoshin.kang@knu.ac.kr; 4 Department of Psychiatry, Seoul National University College of Medicine, Seoul 07061, Republic of Korea

**Keywords:** anxiety, major depressive disorder, bipolar disorder, network analysis, centrality, network structure

## Abstract

*Background and Objectives:* Anxiety is prevalent in patients with major depressive disorder (MDD) and bipolar disorder (BD). Understanding its interaction with mood disorders may provide deeper insight into symptom clustering, severity, and interventions. We compared the networks of MDD and BD using the Beck Anxiety Inventory (BAI) to identify central symptoms and interconnections. *Materials and Methods:* This cross-sectional study involved 815 individuals with MDD (*n* = 332) and BD (*n* = 483) who had clinically significant anxiety symptoms (BAI score > 8). Network analysis identified anxiety symptom clusters. Network centrality, stability, and comparison tests assessed the structural differences and global strength variations between the groups. *Results:* Both the MDD and BD networks showed strong interconnections among several BAI items and demonstrated stable centrality measures. Core symptoms with high centrality included “Losing control”, “Choking”, “Breathing”, “Unsteady”, and “Shaky” in both MDD and BD. Although no significant differences were found in the overall network structures between MDD and BD, the global strength of the network differed significantly, with MDD exhibiting modestly higher overall anxiety network connectivity than BD. *Conclusions:* Network clusters revealed aspects of both cognitive and somatic symptoms of anxiety. Although overall structures were similar between the groups, the MDD group showed stronger interconnections for central anxiety symptoms. Targeting central anxiety symptoms can enhance prevention and intervention strategies for mood disorders and improve clinical outcomes.

## 1. Introduction

Mood disorders encompass a wide array of disorders that affect emotions, energy levels, and motivation; such effects are prominent features of major depressive disorder (MDD) and bipolar disorder (BD) [1]. MDD and BD impact quality of life and psychosocial functioning [2,3,4,5]. Furthermore, mood disorders are associated with increased suicide rates and mortality [3,4,5], and are comorbid with other mental health disorders, including anxiety, substance use, and personality disorders [6,7,8,9].

Anxiety symptoms are common in patients with mood disorders, with comorbidity rates of 45.7% in MDD and 51.2% in BD [10,11]. Studies have demonstrated a high comorbidity of anxiety symptoms in patients with mood disorders [12]. Anxiety symptoms commonly occur in mood disorders as central symptoms (e.g., worry, nervousness, and panic) and somatic symptoms (e.g., restlessness, sweating, and dizziness) [11]. Mood disorders with anxiety symptoms can cause discomfort and increase symptom severity [13]. Therefore, it is essential to understand the symptoms of mood and anxiety disorders to improve treatment outcomes in patients [14].

Network perspectives on symptoms strengthen the understanding of psychopathology and etiology by connecting the co-occurrence and interactions between symptoms and disorders [15,16]. The nodes in network analysis show how symptoms are more strongly connected in the network. The edges represent links between symptoms in the network. This demonstrates the significance of the relationship between symptoms [17]. Specifically, network analysis helps identify unique relationships between symptoms to develop targeted interventions based on the strength and complexity of the network structure [18,19]. The strength centrality has been used as one of the most robust and reliable centrality indices in psychological network analyses [20].

Network analysis has been used in various studies on mood and anxiety disorders [18,21,22]. Previous studies using network analysis examined a wide range of psychiatric symptoms, including anxiety, psychosis, substance use, posttraumatic stress, and depressive and manic symptoms [18,23,24]. A study that focused on mood disorders and network analysis of anxiety symptoms identified that the inability to relax may be related to depression in people who have been exposed to traumatic events [21]. A recent network analysis study in middle-aged and older adults with comorbid anxiety and depression demonstrated network connections of somatic anxiety symptoms (e.g., restlessness and psychomotor agitation) and cognitive symptoms (e.g., concentration problems), highlighting bridge symptoms and capturing cross-domain interactions between anxiety and depression [25]. Furthermore, certain anxiety symptoms (e.g., feeling shaky, fear, and faintness) were identified as central anxiety symptoms in people with major depression [21]. However, previous studies have not fully elucidated either the network structures of anxiety symptoms in major depressive disorder (MDD) and bipolar disorder (BD) or their differences, leaving gaps in the understanding of the distinct anxiety symptomatology of each disorder.

In this study, we evaluated the anxiety symptoms in patients with MDD and BD using network analysis. Specifically, we aimed to (1) identify the central symptoms and distinct communities of interconnected anxiety symptoms across mood disorders in an adult population, and (2) compare the network structure between patients with MDD and BD (including all participants with BD I and BD II). We additionally examined the robustness of network findings among individuals with depressive symptoms using sensitivity analysis.

## 2. Methods

### 2.1. Participants and Ethical Statement

A total of 815 participants (MDD: *n* = 332, BD I: *n* = 99, BD II: *n* = 384) were enrolled in this study. Individuals who scored < 8 on the Beck Anxiety Inventory (BAI) were excluded to concentrate on the significant anxiety symptoms within the MDD and BD groups, thereby excluding those with minimal anxiety symptoms. On the BAI, a score of 8 or higher is generally considered to indicate at least mild clinical anxiety [26,27]. All diagnoses were made by board-certified psychiatrists (W.M. and T.H.H.) through structured diagnostic interviews using the Mini-International Neuropsychiatric Interview (M.I.N.I) [28], a comprehensive review of case records, or other available data. Diagnoses were made according to the criteria specified in the DSM-5 [29]. Demographic data and information related to anxiety symptom scales were collected from the patients. Participants received routine pharmacological treatment according to clinical judgment. This study complied with the ethical rules stated in the Declaration of Helsinki. The study procedures were approved by the Institutional Review Board of Seoul National University Bundang Hospital. The study data were acquired from the medical records; therefore, the collection of informed consent forms was waived. The requirement for comparison consent was also waived because the researchers did not have direct access to the participants’ personal information and used anonymized survey data for the analyses.

### 2.2. Measurements

#### Beck Anxiety Inventory

The BAI is a 21-item self-reported questionnaire that assesses the severity of anxiety symptoms experienced in the past week [30]. This study used the Korean version of the BAI, which has been validated for its reliability and consistency [31]. Responses are rated on a scale of 0 (not at all) to 3 (severely). The total score ranges from 0 to 63, with higher scores indicating more significant anxiety levels. Clinical classification based on scoring results is as follows: 0–7 indicates minimal anxiety, 8–15 suggests mild anxiety, 16–25 suggests moderate anxiety, and 26–63 suggests severe anxiety [26,27]. The BAI demonstrated high internal reliability and good factorial and discriminant validity [32]. The BAI was developed to assess anxiety symptoms across a range of psychiatric disorders, including both MDD and BD [33,34].

### 2.3. Statistical Analysis

A network was constructed and visualized using a Gaussian graphical model [35]. In the network, the nodes represent items from the BAI, and the edges denote the bivariate partial correlation coefficients between these items [36]. Within the network structure, nodes exhibiting greater connectivity and stronger correlations are centralized, whereas nodes with fewer and weaker connections are positioned at the periphery. Meanwhile, the thickness of the edge indicates the magnitude of the correlation between nodes. To enhance the network’s stability and reliability by reducing spurious edges, the graphical least absolute shrinkage and selection operator (glasso) method was employed. The EBICglasso function from the “qgraph” R package (version 1.9.8; using R Foundation for Statistical Computing version 4.2.2.) was used to conduct this analysis [37,38].

An exploratory graph analysis (EGA), a data-driven community detection method, was used to identify distinct communities of items within the network based on the inherent dimensionality of the items [39,40,41]. The Louvain algorithm was applied to determine the optimal community structure within the BAI items, which focus on optimizing the hierarchically structured modularity measure among vertices within these communities [42]. A parametric bootstrapping procedure was implemented to enhance the robustness of the EGA findings [43], utilizing the EGA and bootEGA functions from the “EGAnet” R package (version 2.0.0; R Foundation for Statistical Computing version 4.2.2) [44,45].

To identify and evaluate the centrality of symptoms in patients with MDD and BD, several centrality indices were employed: (1) strength centrality (SC), which was calculated as the sum of all direct association weights for a specific item. This index is recognized for its reliability and stability within symptom networks, where nodes with higher strength centrality exert greater influence on the entire network; (2) closeness centrality (CC), which reflects the frequency at which a node appears on the shortest indirect path relative to another node; and (3) betweenness centrality (BC), which quantifies the extent to which an item mediates the interaction between other items [46]. Given prior evidence that node strength tends to be the most stable and interpretable centrality index in psychological symptom networks, our primary interpretations therefore focused on strength centrality rather than on closeness or betweenness centrality [38].

Network stability and the integrity of the centrality measures were evaluated using the correlation stability coefficient (CS coefficient), which indicates the maximum proportion of cases that can be excluded while retaining a correlation of at least 0.7 with the original dataset, with a confidence level of 95% [38]. A CS-coefficient value > 0.25 is indicative of moderate stability, whereas a value > 0.50 denotes strong stability [38]. Bootstrapped difference tests were conducted to assess the differences in strength centrality and edge weights among all the node connections. The significance of these differences was evaluated using 95% bootstrap confidence intervals (CIs), where narrow CIs indicate precise estimations and CIs that encompass zero suggest there is no significant variance between nodes. The “bootnet” R package (version 1.5.6; R Foundation for Statistical Computing version 4.2.2) was used to implement these analyses [35].

The network structures of the MDD and BD cohorts were compared using Network Comparison Tests (NCTs), regarding the structural differences (network invariance), number of edges with significant differences (edge invariance), and overall network connectivity (global strength). To adjust for the risk of type I errors from multiple comparisons, a Holm–Bonferroni correction was applied [47]. These analyses were conducted using the NCT package.

In addition, because the expression of anxiety may vary across the current mood states of patients, we conducted an additional sensitivity analysis focusing on participants with more pronounced depressive symptomatology. Specifically, we repeated the network analyses in a subsample restricted to participants with a Zung Self-Rating Depression Scale (SDS) score of ≥50, approximating at least mild depressive symptoms [48]. This subsample consisted of 607 individuals (MDD: *n* = 233; BD: *n* = 374). Within this subsample, separate networks were estimated for the MDD and BD groups, and community detection and centrality indices were performed using the same procedures as described above.

## 3. Results

### 3.1. Demographic and Clinical Characteristics

Table 1 presents the demographic characteristics of patients diagnosed with MDD and BD. The mean age of the cohort was 34.85 (SD = 12.66) years, with a predominance of female participants (70.7%). The results showed significant differences between the MDD and BD groups in terms of age, marital status, and family psychiatric history, after Bonferroni correction. Patients with BD were typically younger, less likely to be married, and had a more prevalent family psychiatric history than those with MDD. In contrast, there were no significant between-group differences in gender, education level, employment status, alcohol use, or smoking status after Bonferroni correction. In terms of depressive symptom severity, the distribution of Zung SDS scores indicated that the majority of participants were at least mildly depressed at the time of assessment: approximately three-quarters of the total sample (607/815) scored 50 or higher, with similar proportions in the MDD and BD groups. Table 2 presents the item-level BAI scores for the MDD and BD groups. After applying Bonferroni correction, neither the total BAI score nor any individual BAI items showed significant between-group differences, indicating that overall anxiety severity and item-level symptom profiles were broadly comparable across the two groups.

### 3.2. Network and Community Estimation

**Figure 1:**
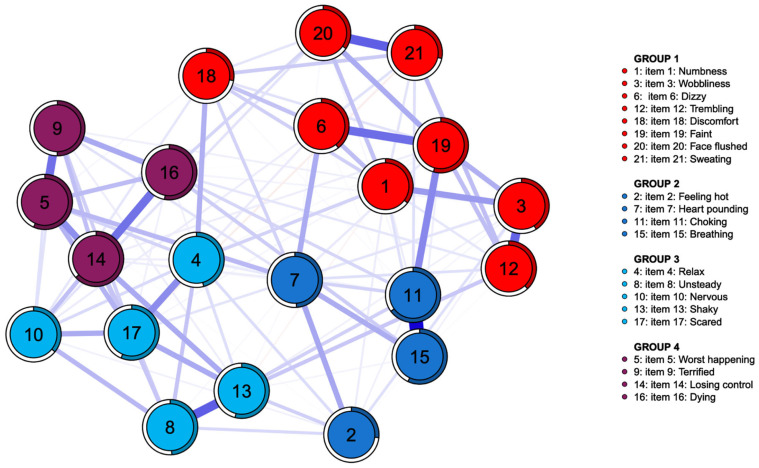
Symptom network structure of the Beck Anxiety Inventory in major depressive disorder (*n* = 332). Node colors represent four symptom communities identified by exploratory graph analysis (EGA).

**Figure 2:**
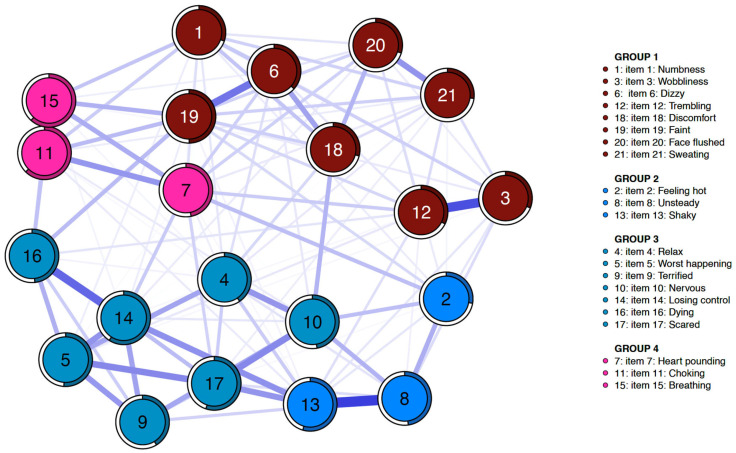
Symptom network structure of the Beck Anxiety Inventory in bipolar disorder (*n* = 483). Node colors represent four symptom communities identified by exploratory graph analysis (EGA).

Figure 1 and Figure 2 illustrate the estimated symptom networks for the MDD and BD groups, respectively. Four distinct communities were identified for each diagnostic group. In the MDD cohort, Group 1 consisted of items 1 (“Numbness or tingling”), 3 (“Wobbliness in legs”), 6 (“Dizzy or lightheaded”), 12 (“Hands trembling”), 18 (“Indigestion”), 19 (“Faint”), 20 (“Face flushed”), and 21 (“Hot/cold sweats”); Group 2 of items 2 (“Feeling hot”), 7 (“Heart pounding/racing”), 11 (“Feeling of choking”), and 15 (“Difficulty in breathing”); Group 3 of items 4 (“Unable to relax”), 8 (“Unsteady”), 10 (“Nervous”), 13 (“Shaky”), and 17 (“Scared”); and Group 4 of items 5 (“Fear of worst happening”), 9 (“Terrified or afraid”), 14 (“Fear of losing control”), and 16 (“Fear of dying”). Whereas, in the BD cohort, Group 1 included items 1 (“Numbness or tingling”), 3 (“Wobbliness in legs”), 6 (“Dizzy or lightheaded”), 12 (“Hands trembling”), 18 (“Indigestion”), 19 (“Faint”), 20 (“Face flushed”), and 21 (“Hot/cold sweats”); Group 2, items 2 (“Feeling hot”), 8 (“Unsteady”), and 13 (“Shaky”); Group 3, items 4 (“Unable to relax”), 5 (“Fear of worst happening”), 9 (“Terrified or afraid”), 10 (“Nervous”), 14 (“Fear of losing control”), 16 (“Fear of dying”), and 17 (“Scared”); and Group 4, items 7 (“Heart pounding/racing”), 11 (“Feeling of choking”), and 15 (“Difficulty in breathing”). Bootstrapped iterations revealed that the four-community model had a replication rate of 61.0% (610 out of 1000) in the MDD group and 65.3% (653 out of 1000) in the BD group, indicating relatively high replication rates and moderate stability of the identified community structures. Although some items shifted between communities across the two diagnostic groups, these differences in community allocation appeared modest and primarily involved somatic or closely related cognitive symptoms. Bootstrapped node- and edge-level difference tests for these community structures are presented in Supplementary Figures S1–S4.

### 3.3. Centrality Indices and Edge Weights

**Figure 3:**
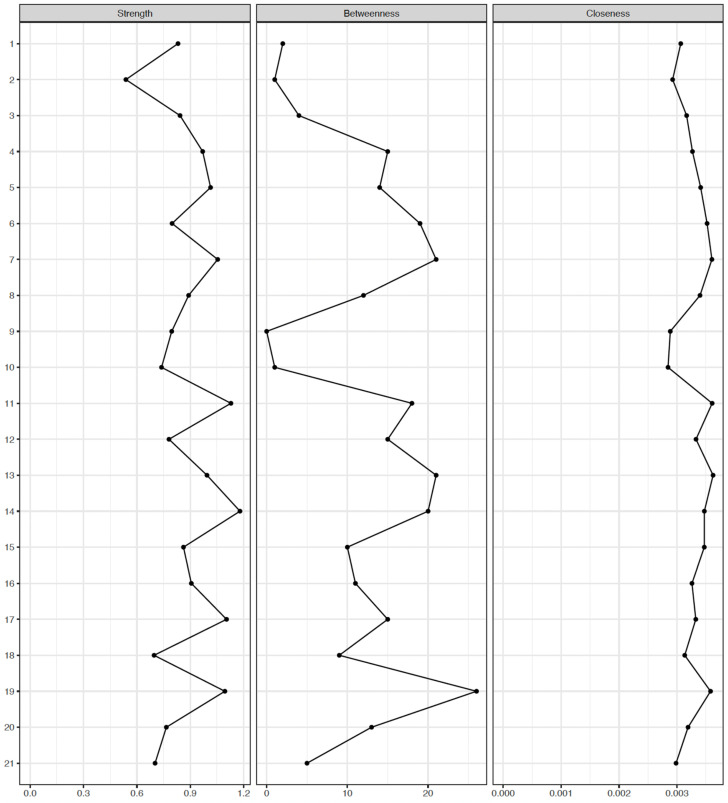
Centrality indices of Beck Anxiety Inventory symptoms in major depressive disorder (*n* = 332). Strength centrality was treated as the primary and most reliable index of symptom importance, given the lower stability of betweenness and closeness centrality in the bootstrap analyses.

**Figure 4:**
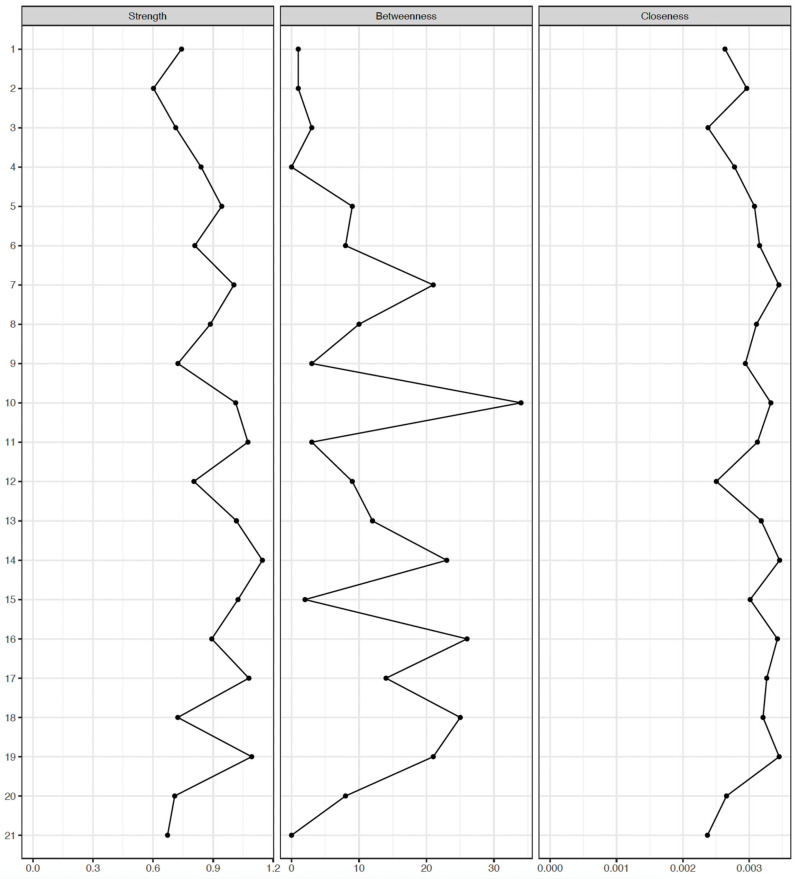
Centrality indices of Beck Anxiety Inventory symptoms in bipolar disorder (*n* = 483). Strength centrality was treated as the primary and most reliable index of symptom importance, given the lower stability of betweenness and closeness centrality in the bootstrap analyses.

Centrality indices are outlined in Figure 3 and Figure 4, with item 14 (“Losing control”) consistently showing the highest strength centrality for both MDD and BD groups (strength = 1.179, betweenness = 40, and closeness = 0.003 for the MDD group; strength = 1.145, betweenness = 46, and closeness = 0.0034 for the BD group). In the MDD group, items 11 (“Choking”), 17 (“Scared”), 19 (“Faint”), and 7 (“Heart pounding”) followed in terms of strength centrality, whereas, items 19 (“Faint”), 17 (“Scared”), 11 (“Choking”), 15 (“Breathing”), and 13 (“Shaky”) followed in the BD group. No significant differences in strength centrality were observed among items 14, 11, 17, 19, and 7 in the MDD group and items 14, 19, 17, 11, 15, and 13 in the BD group. Further, bootstrapped tests for edge weights (Supplementary Figures S3 and S4) showed that the edges between items 11 (“Choking”) and 15 (“Breathing”) and items 8 (“Unsteady”) and 13 (“Shaky”) were most prominent in both groups. Given that strength centrality demonstrated the highest stability in the bootstrap analyses (Supplementary Figures S5 and S6), strength centrality was considered the most informative index, and the clinical interpretation of symptom importance primarily relied on strength rather than betweenness or closeness centrality [38].

### 3.4. Network Stability

Network stability was evaluated using centrality indices of strength, betweenness, and closeness for both the MDD and BD groups (Supplementary Figures S5 and S6). The analytical outcomes demonstrated strong stability in terms of strength centrality, with CS-coefficients exceeding 0.5 (CS coefficient = 0.518 for the MDD group and 0.673 for the BD group). Meanwhile, the betweenness and closeness centrality indices showed moderate-to-low stability, with CS coefficients of 0.127 for both betweenness and closeness in the MDD group, and 0.125 for betweenness and 0.284 for closeness in the BD group.

### 3.5. Network Comparisons

A comparative network analysis was conducted between the MDD and BD groups (MDD: *n* = 332, BD I: *n* = 99, BD II: *n* = 384). It revealed no significant differences in the overall network structure between the two groups (M = 0.12, n.s.). However, a significant difference was observed in the global strengths of the networks (MDD = 9.879, BD = 9.321; S = 0.558, *p* = 0.027). Furthermore, the edge and centrality invariance comparison tests did not reveal any statistically significant differences.

### 3.6. Sensitivity Analysis

To examine the robustness of the findings among patients with more pronounced depressive symptoms, we repeated the network analyses in a subsample restricted to participants with a Zung SDS score ≥ 50 (MDD: *n* = 233; BD: *n* = 374). In the MDD group, the estimated symptom network and community structure in this subsample were virtually identical to those observed in the full sample: four communities were again identified, with Beck Anxiety Inventory (BAI) items clustering into somatic, cardiopulmonary, and fear-related groups in a similar pattern, and item 14 (“Losing control”) showed the highest strength centrality, followed by items such as 11 (“Feeling of choking”), 17 (“Scared”), 19 (“Faint”), and 7 (“Heart pounding/racing”), as in the main analysis (Supplementary Figures S7 and S8).

In the BD group, exploratory graph analysis in the Zung SDS score ≥ 50 subsample yielded a slightly more compact three-community solution rather than the four-community solution observed in the full sample. Nevertheless, somatic items (e.g., items 1 “Numbness or tingling”, 3 “Wobbliness in legs”, 6 “Dizzy or lightheaded”, 12 “Hands trembling”, 18 “Indigestion”, 19 “Faint”, 20 “Face flushed”, 21 “Hot/cold sweats”), cardiopulmonary items (e.g., items 2 “Feeling hot”, 7 “Heart pounding/racing”, 11 “Feeling of choking”, 15 “Difficulty in breathing”), and fear-related cognitive items (e.g., items 5 “Fear of worst happening”, 9 “Terrified or afraid”, 14 “Fear of losing control”, 16 “Fear of dying”, 17 “Scared”) continued to form coherent clusters, indicating that the overall organization of somatic and cognitive anxiety dimensions was preserved despite the reduction in the number of communities (Supplementary Figure S7). The pattern of strength centrality in the BD subsample also closely resembled the main analysis: item 14 (“Losing control”) again showed the highest strength centrality, and somatic and cardiopulmonary items remained among the most central BAI items (Supplementary Figure S8).

## 4. Discussion

The present study investigated the network structure of anxiety symptoms in individuals with major depressive disorder (MDD) and bipolar disorder (BD). We conducted a network analysis using the BAI with a four-community model to identify anxiety symptom networks and distinct communities of interconnected symptoms. One of our main findings was that “Losing control (item 14)” exhibited the highest centrality strength in both MDD and BD groups. Furthermore, certain symptom pairs, such as “Choking (item 11)” and “Breathing (item 15)” and “Unsteady (item 8)” and “Shaky (item 13)”, demonstrated robust network connections across both groups.

In interpreting centrality results, it is notable that strength centrality provides a reliable index in psychological symptoms, with higher strength centrality reflecting stronger connections with other symptoms within the network [49]. The overall network structures of MDD and BD were similar, with no significant differences found in the network comparison test [5]. Although no significant differences were found in overall network structure, we observed a statistically significant difference in global strength between the MDD and BD groups from NCT. The absolute difference in global strength was modest (MDD = 9.879, BD = 9.321; S = 0.558), suggesting a small increase in overall symptom connectivity in the MDD network. This should be interpreted with caution, as the finding likely reflects a subtle difference in overall symptom connectivity rather than distinct network structures between MDD and BD. Our results suggest that anxiety symptoms in MDD and BD share underlying pathways, providing further insight into the prevalence, risk, and severity of anxiety-related outcomes in mood disorders, consistent with previous studies [17,21,43].

In the MDD group, significant strength of the centrality of BAI items was noted: “Choking (item 11),” “Scared (item 17),” “Faint (item 19),” and “Heart pounding (item 7).” This finding aligns with a previous study, indicating that feelings of choking, fear, and faintness, derived from the BAI, were noted as central anxiety symptoms in patients with MDD [21]. Understanding these central anxiety symptoms is crucial for improving symptom monitoring, assessing risk factors, and identifying potential comorbidities [24]. Furthermore, the network analysis of central symptoms can offer deeper insights into the severity, potential causality of symptoms, and development of interventions for mood disorders [50]. It can also contribute to the prevention of mood disorders and predict relapse in patients at high risk [50].

We also identified strong network connections among somatic symptoms in the BAI. Specifically, “Choking (item 11)” was strongly linked to “Breathing (item 15),” while “Unsteady (item 8)” showed robust connectivity with “Shaky (item 13)” in both the MDD and BD groups. Previous studies have suggested that stronger connectivity of somatic symptoms (e.g., feelings of shakiness and dizziness) can impact treatment response, remission rates, and overall outcomes in patients with mood disorders [51,52]. The co-occurrence of somatic symptoms heightens the severity of mood disorders by increasing psychophysiological distress [52,53,54]. Our findings highlight the need for further research on how these interconnections function within the BAI network in mood disorders [55]. Future research should aim to provide valuable clinical insights into mood disorders that are frequently comorbid with anxiety symptoms.

Multiple studies have reported associations between the BAI and mood disorders using network analysis [18,22,56,57]. However, most studies did not identify significant differences in global strength after NCT. In the current study, NCT did not indicate significant differences between MDD and BD, except for global strength. A previous study on acute depression across MDD and BD was unable to find notable data from the NCT in terms of network structure, strength, or edge correlations [56]. These similarities suggest that the anxiety symptom networks in MDD and BD share comparable underlying structures. Furthermore, indicating the severity of symptoms in MDD and BD may reflect on symptom presentation and assessment [56].

Clinically, these findings imply that interventions targeting central anxiety symptoms may be effective in both MDD and BD populations. Our analysis of global strength revealed a significant trend, suggesting that the overall connectivity of anxiety symptoms within the MDD and BD groups may differ in terms of intensity [58,59]. The expression of anxiety symptoms in BD can vary across mood states (e.g., depressive, manic, hypomanic, or euthymic), which can reflect overall network connectivity [60]. Understanding shared central symptoms can benefit clinicians, who may be better equipped to develop cross-diagnostic treatment strategies that address the overlapping anxiety dimensions, potentially improving outcomes for patients with mood disorders.

Our findings indicate that central anxiety symptoms identified through the network analysis of the BAI in mood disorders included both cognitive and somatic symptoms. Cognitive symptoms refer to distressing thoughts and fears (e.g., excessive worry, fear of losing control), while somatic symptoms involve physical sensations (e.g., dizziness, shortness of breath) [61,62]. Both mood disorders showed the highest strength in “Losing control (item 14).” The observed connectivity between somatic and cognitive anxiety symptoms in our network may reflect broader physiological dysregulation in mood disorders. A study suggested that abnormalities in neural and physiological systems, especially those involved in arousal, sleep–wake cycle, inflammatory system, and fatigue, are related to cognitive dysfunction in psychiatric disorders [63]. Such physiological dysregulation may contribute to increasing somatic sensitivity and cognitive dysfunction, which can help explain the somatic symptoms and anxiety cluster observed in our network analysis.

This study has several limitations that should be considered when interpreting the findings. The use of the BAI as a self-report measure introduces potential bias due to the subjective nature of the responses. Self-reported data may be influenced by individual differences in the perception and interpretation of anxiety symptoms, which may affect the reliability of the results. A further limitation includes the exclusive use of the BAI to analyze the anxiety symptom network. The BAI is primarily oriented toward somatic and panic-related symptoms and places less emphasis on cognitive symptoms of anxiety, such as excessive worry or fear anticipation. As a result, the observed network structure of the BAI may partly reflect the network structure of the measurement itself, rather than the broader anxiety symptoms in mood disorders. To more comprehensively capture anxiety symptoms, future studies may consider using additional anxiety measures such as the Generalized Anxiety Disorder-7, which assesses the severity of anxiety that includes cognitive symptoms like excessive worry and irritability [64]. Furthermore, the participants were recruited solely from a university-affiliated hospital, which limits the generalizability of our findings. The sample may not be representative of the broader population, as it lacks diversity in terms of socioeconomic, geographical, and clinical backgrounds.

In addition, we did not systematically incorporate other clinical variables that may shape anxiety symptom networks, such as illness duration, comorbid psychiatric diagnoses (e.g., comorbid anxiety disorders), or detailed information on current pharmacological treatment (e.g., benzodiazepines, antidepressants, mood stabilizers). Thus, differences in network structure between MDD and BD could partly reflect medication patterns or comorbidity profiles rather than diagnostic category alone. The exclusion of participants with low levels of anxiety (BAI score < 8) may limit the generalizability of the findings and restrict the range of symptom variability in the network. Another limitation concerns the characterization of the bipolar subsample. Because the number of patients with BD I was relatively small, network models could not be robustly estimated for BD I and BD II separately, and these subtypes were therefore combined into a single BD group. Moreover, although depressive symptom severity was assessed using the Zung SDS, systematic information on current mood episode type in BD (e.g., depressive, hypomanic/manic, mixed, euthymic) was not available, which constrains the interpretation of anxiety networks across different phases of bipolar disorder. To enhance generalizability, future studies should include participants from a wide range of clinical settings and demographic backgrounds. This study used a cross-sectional design, which prevents causal inferences regarding the relationships between anxiety symptoms. It remains unclear whether the central symptoms identified in the network analysis exert a causal influence on other symptoms or whether they are consequences of broader symptom clusters. Longitudinal studies are necessary to examine the directional relationship between the stability of anxiety symptoms in mood disorders.

## 5. Conclusions

This study conducted a network analysis to examine how various anxiety symptoms manifest in mood disorders. Despite its limitations, including the cross-sectional design and reliance on a self-report measure, this study provides valuable insights into the network structure of anxiety symptoms in patients with MDD and BD (including both BD I and BD II). Our findings highlight potential intervention targets by identifying central symptoms and robust networks. Clinically, targeting symptoms with a high centrality can improve treatment outcomes by disrupting key pathways within the symptom network. Differences in global strength between MDD and BD should be interpreted cautiously, as clinical heterogeneity and mood-state variability within bipolar disorder may influence overall network connectivity results. Furthermore, recognizing the shared symptom structure of MDD and BD may facilitate the development of cross-diagnostic treatment strategies that address overlapping anxiety features and ultimately enhance the effectiveness of interventions for individuals with mood disorders. Future research should explore how targeting these central symptoms may affect treatment responses and recovery trajectories, particularly using longitudinal and multimodal approaches to clarify how symptom networks and central anxiety symptoms evolve over time and to optimize anxiety comorbid with mood disorders.

## Figures and Tables

**Table 1:** Clinical and demographic characteristics of all participants (*n* = 815).

Characteristics	Total Sample (*n* = 815) Mean ± SD, *n* or %	MDD (*n* = 332) Mean ± SD, *n* or %	BD (*n* = 483) Mean ± SD, *n* or %	Test Statistics	Bonferroni Corrected *p*-Value
Age (years)	34.85 ± 12.66	38.53 ± 13.26	32.34 ± 11.59	7.0452	<0.001 ***
Gender (%)				1.25	1.00
Male	29.3	31.6	27.7		
Female	70.7	68.4	72.3		
Education (%)				1.10	1.00
High school or below	29.4	31.6	28.0		
Others	70.6	68.4	72.0		
Employment status (%)				0.33	1.00
Unemployed	63.7	62.3	64.6		
Employed	36.3	37.7	35.4		
Marital status (%)				21.0294	<0.001 ***
Married	35.5	44.9	29.0		
Others (Single, divorced, or widowed)	64.5	55.1	71.0		
Alcohol use status (%)				6.2666	0.098
Former or current	55.7	50.3	59.4		
Never	44.3	49.7	40.6		
Smoking status (%)				3.0244	0.656
Past or current	28.5	25.0	30.8		
Never	71.5	75.0	69.2		
Psychiatric familial history	44.8	33.7	52.4	26.9147	<0.001 ***
Zung Self rating depression scale score	55.28 ± 9.00	54.92 ± 9.58	55.53 ± 8.58	−0.93669	1.00
Range (*n*)					
<50	208	99	109		
50 ≤ *x* < 60	310	115	195		
60 ≤ *x* < 70	270	107	163		
≥70	27	11	16		

Note. *** *p* < 0.001.

**Table 2:** Analysis of item scores of Beck Anxiety Inventory (BAI) between major depressive disorder (MDD) and bipolar disorder (BD).

	Items	Mean (SD)		U_Statistic	*p*-Value	Bonferroni Corrected
		MDD (*n* = 332)	BD (*n* = 483)			*p*-Value
1	Numbness or tingling	1.16 (0.98)	1.06 (0.95)	84,790.5	0.14	1.00
2	Feeling hot	0.99 (0.91)	1.03 (0.93)	78,216	0.53	1.00
3	Wobbliness in legs	0.72 (0.83)	0.77 (0.84)	76,879.5	0.28	1.00
4	Unable to relax	1.64 (0.94)	1.61 (0.97)	81,104.5	0.77	1.00
5	Fear of worst happening	1.46 (0.96)	1.56 (1.00)	75,714	0.16	1.00
6	Dizzy or lightheaded	1.20 (0.94)	1.17 (0.94)	80,992.5	0.80	1.00
7	Heart pounding/racing	1.47 (0.88)	1.46 (0.96)	81,205.5	0.74	1.00
8	Unsteady	1.36 (0.87)	1.42 (0.90)	76,784.5	0.27	1.00
9	Terrified or afraid	1.31 (0.96)	1.39 (0.97)	77,055.5	0.32	1.00
10	Nervous	1.75 (0.89)	1.72 (0.94)	81,274.5	0.73	1.00
11	Feeling of choking	1.05 (1.02)	1.06 (1.02)	79,616.5	0.86	1.00
12	Hands trembling	0.90 (0.95)	1.03 (1.02)	74,967	0.10	1.00
13	Shaky	1.23 (0.93)	1.35 (0.97)	74,409.5	0.07	1.00
14	Fear of losing control	1.19 (1.03)	1.21 (1.08)	79,573	0.85	1.00
15	Difficulty in breathing	0.99 (0.99)	1.06 (1.01)	77,491	0.39	1.00
16	Fear of dying	0.91 (0.92)	0.96 (1.04)	78,531.5	0.6	1.00
17	Scared	1.76 (0.89)	1.77 (0.92)	79,347	0.79	1.00
18	Indigestion	1.37 (0.96)	1.30 (0.98)	83,617	0.28	1.00
19	Faint	0.59 (0.88)	0.61 (0.87)	78,025.5	0.46	1.00
20	Face flushed	0.76 (0.88)	0.76 (0.91)	81,235	0.73	1.00
21	Hot/cold sweats	0.83(0.96)	0.82 (0.94)	80,569.5	0.90	1.00
	Total score	24.63 (12.21)	25.11 (12.35)	78,388	0.59	1.00

## Data Availability

The datasets used and/or analyzed in the current study are available from the corresponding author (WM) upon reasonable request. The data are not publicly available due to the use of patients’ medical information.

## Supplementary Materials

The following supporting information can be downloaded at: https://www.mdpi.com/article/10.3390/medicina61122245/s1, Figure S1. Bootstrapped difference tests between nodes in the BAI 21-items symptoms network among major depressive disorder patients. Figure S2. Bootstrapped difference tests between nodes in the BAI 21-items symptoms network among bipolar disorder patients. Figure S3. Bootstrapped difference tests between edge-weights that were in the BAI 21-items symptoms network among major depressive disorder patients. Figure S4. Bootstrapped difference tests between edge-weights that were in the BAI 21-items symptoms network among bipolar disorder patients. Figure S5. Bootstrapped the centrality stability of the BAI 21-items symptoms network among major depressive disorder patients. Figure S6. Bootstrapped the centrality stability of the BAI 21-items symptoms network among bipolar disorder patients. Figure S7. Symptom network structure of the Beck Anxiety Inventory in major depressive disorder patients with Zung Self-Rating Depression Scale scores ≥ 50 (*n* = 233). Figure S8. Symptom network structure of the Beck Anxiety Inventory in bipolar disorder patients with Zung Self-Rating Depression Scale scores ≥ 50 (*n* = 374). Figure S9. Bootstrapped centrality stability of the BAI 21-item symptom network among major depressive disorder patients with Zung Self-Rating Depression Scale scores ≥ 50 (*n* = 233). Figure S10. Bootstrapped centrality stability of the BAI 21-item symptom network among bipolar disorder patients with Zung Self-Rating Depression Scale scores ≥ 50 (*n* = 374).
