# Supplementary material for: Understanding Anxiety Symptoms of Mood Disorders Across Bipolar and Major Depressive Disorder Using Network Analysis
Source: Medicina (Kaunas). 2025 Dec 18;61(12):2245. doi: 10.3390/medicina61122245 (PMC12734980; doi:10.3390/medicina61122245)

Supplementary Materials

Understanding Anxiety Symptoms of Mood Disorders Across Bipolar and Major Depressive Disorder Using Network Analysis.

Supplementary Materials

Figure S1. Bootstrapped difference tests between nodes in the BAI 21-items symptoms network among major depressive disorder patients.

Figure S2. Bootstrapped difference tests between nodes in the BAI 21-items symptoms network among bipolar disorder patients.

Figure S3. Bootstrapped difference tests between edge-weights that were in the BAI 21-items symptoms network among major depressive disorder patients.

Figure S4. Bootstrapped difference tests between edge-weights that were in the BAI 21-items symptoms network among bipolar disorder patients.

Figure S5. Bootstrapped the centrality stability of the BAI 21-items symptoms network among major depressive disorder patients

Figure S6. Bootstrapped the centrality stability of the BAI 21-items symptoms network among bipolar disorder patients.

Figure S7. Symptom network structure of the Beck Anxiety Inventory in major depressive disorder patients with Zung Self-Rating Depression Scale scores ≥ 50 (n = 233).

Figure S8. Symptom network structure of the Beck Anxiety Inventory in bipolar disorder patients with Zung Self-Rating Depression Scale scores ≥ 50 (n = 374).

Figure S9. Bootstrapped centrality stability of the BAI 21-item symptom network among major depressive disorder patients with Zung Self-Rating Depression Scale scores ≥ 50 (n = 233).

Figure S10. Bootstrapped centrality stability of the BAI 21-item symptom network among bipolar disorder patients with Zung Self-Rating Depression Scale scores ≥ 50 (n = 374).

Figure S1. Bootstrapped difference tests between nodes in the BAI 21-items symptoms network among major depressive disorder patients

Note: Gray boxes indicate that the nodes are not significantly different from each other, while black boxes indicate that the nodes are significantly different (α = 0.05) from each other.

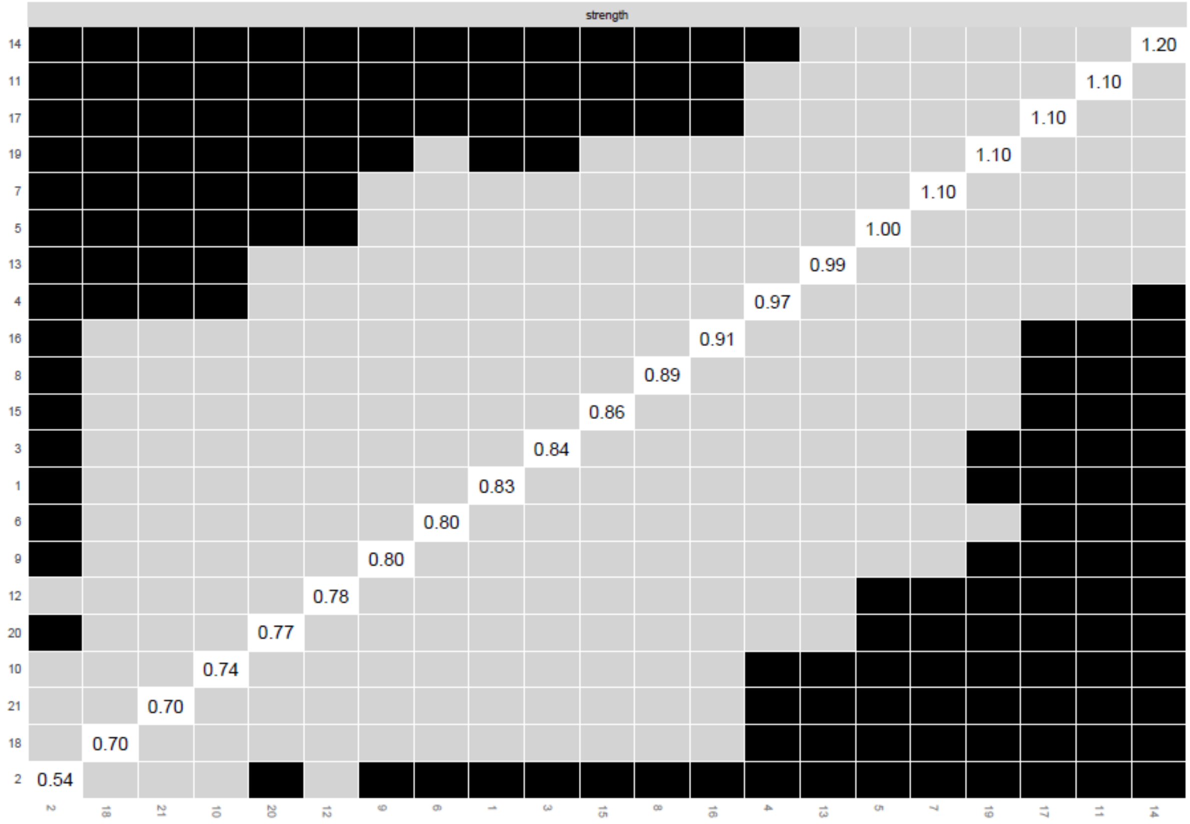

Figure S2. Bootstrapped difference tests between nodes in the BAI 21-items symptoms network among bipolar disorder patients

Note: Gray boxes indicate that the nodes are not significantly different from each other, while black boxes indicate that the nodes are significantly different ($\alpha = 0.05$) from each other.

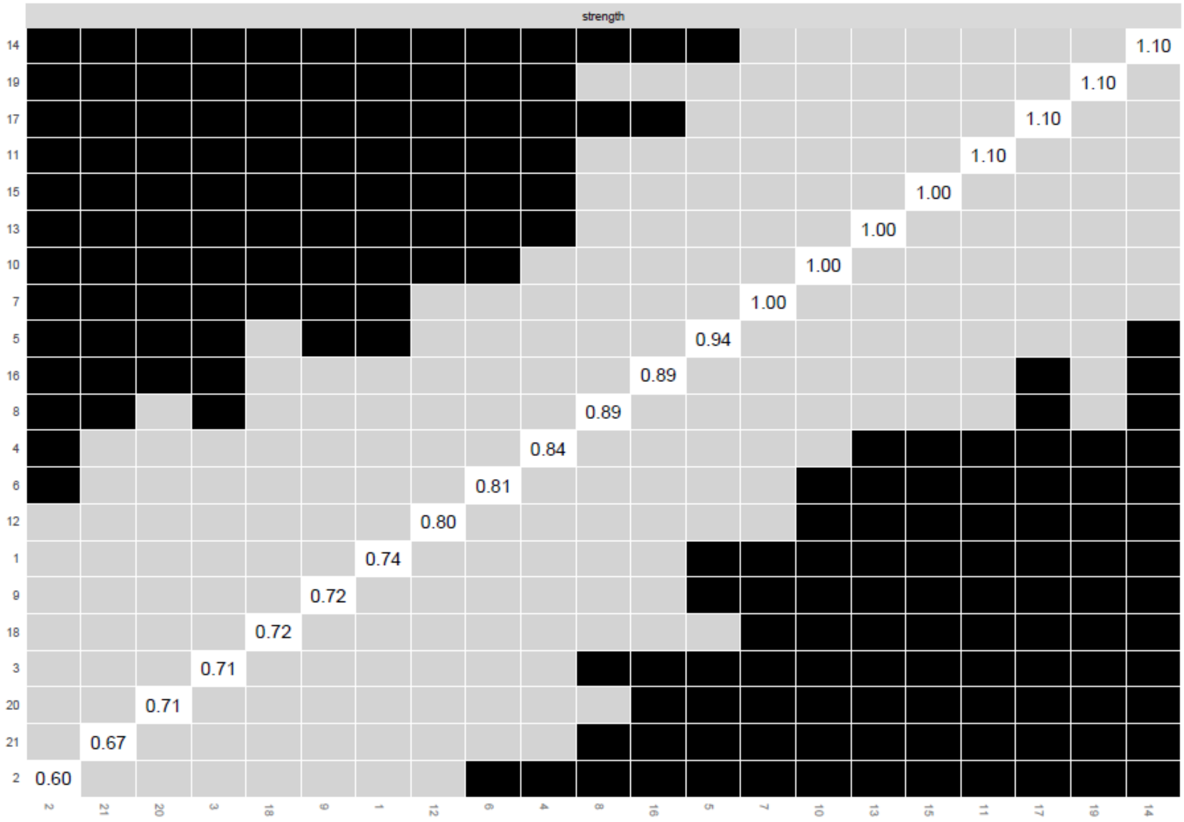

Figure S3. Bootstrapped difference tests between edge-weights that were in the BAI 21-items network among major depressive disorder patients

Note: Gray boxes indicate that the nodes are not significantly different from each other, while black boxes indicate that the nodes are significantly different (α = 0.05) from each other.

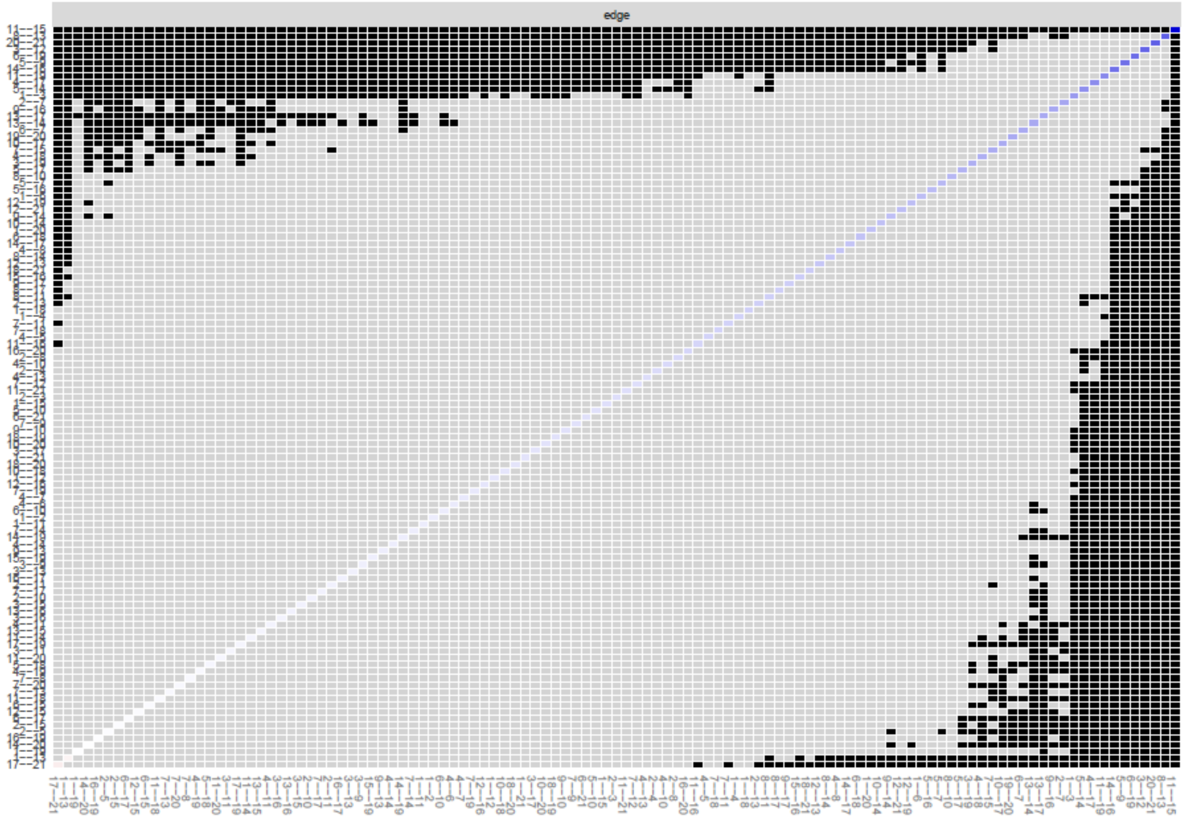

Figure S4. Bootstrapped difference tests between edge-weights that were in the BAI 21-items symptoms network among bipolar disorder patients

Note: Gray boxes indicate that the nodes are not significantly different from each other, while black boxes indicate that the nodes are significantly different (α = 0.05) from each other.

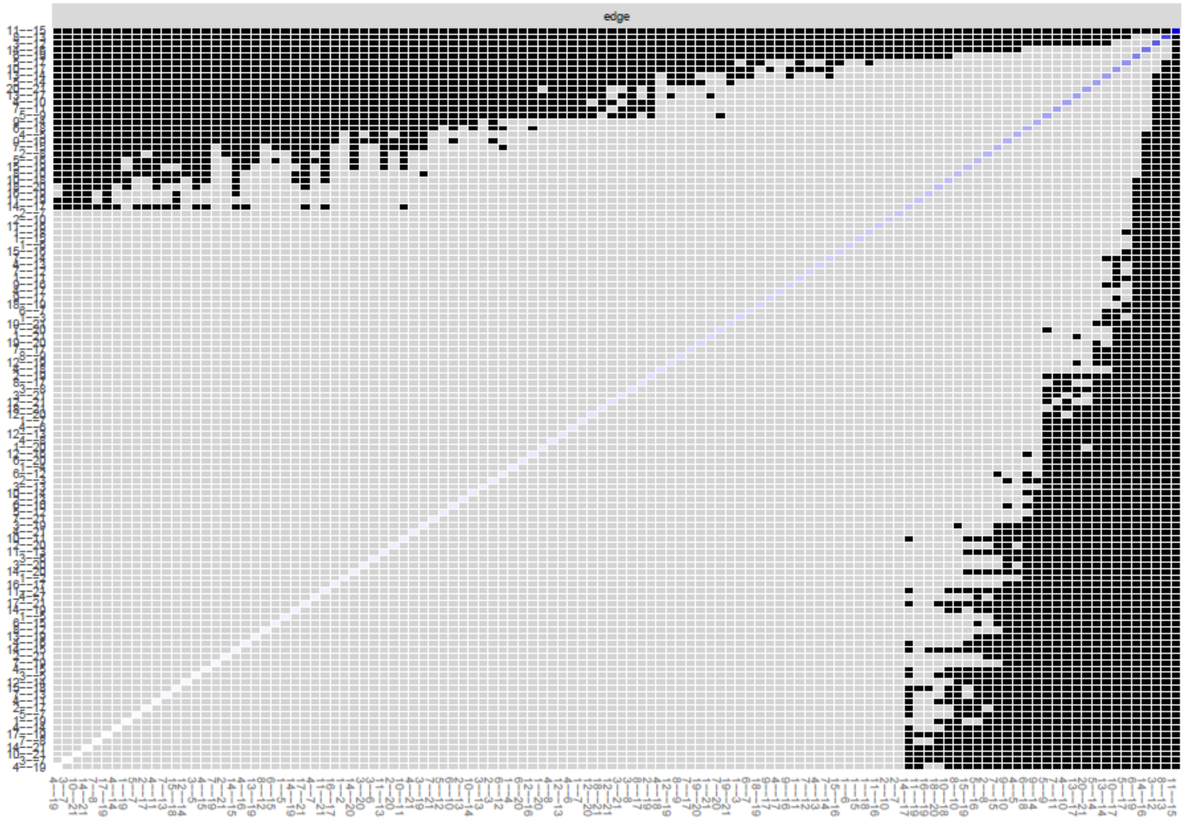

Figure S5. Bootstrapped the centrality stability of the BAI 21-items symptoms network among major depressive disorder patients

Note: The x-axis represents the percentage of cases of the original sample. The y-axis represents the average of correlations between centrality indices in the original sample and sample with case-dropped.

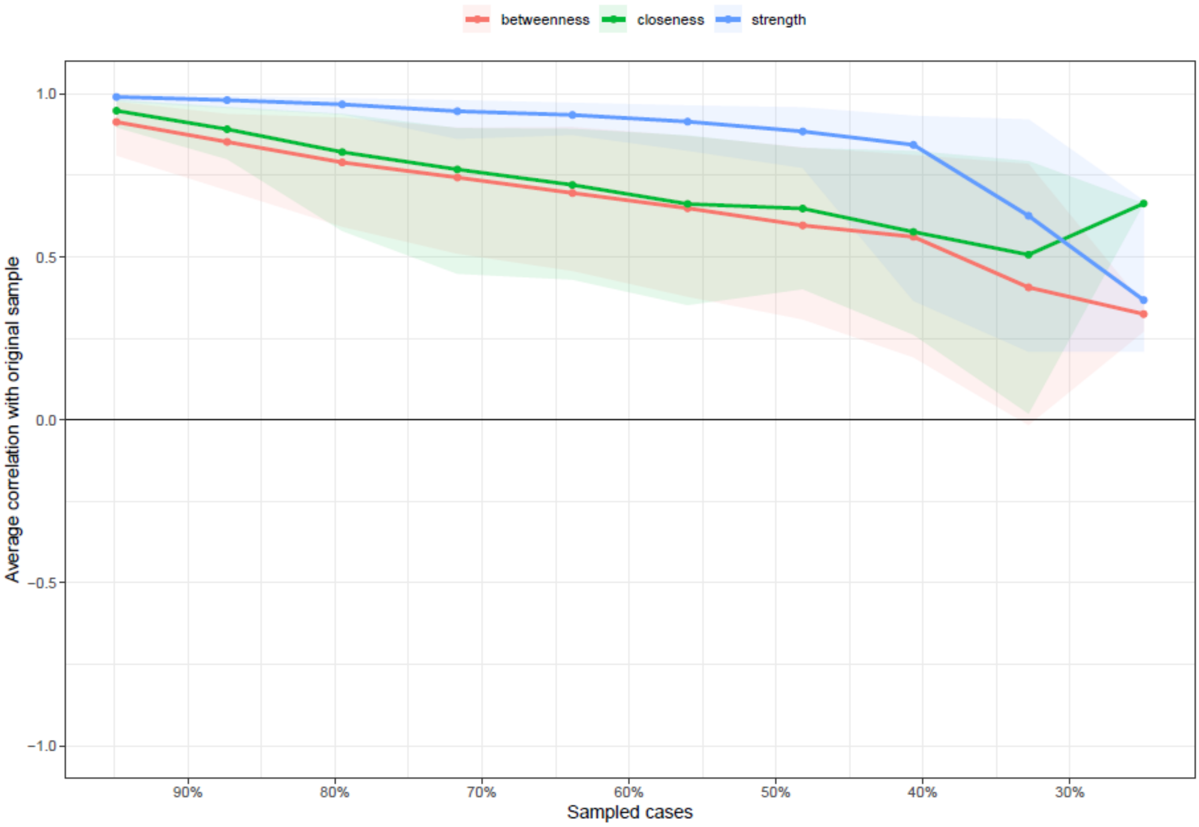

Figure S6. Bootstrapped the centrality stability of the BAI 21-items symptoms network among bipolar disorder patients

Note: The x-axis represents the percentage of cases of the original sample. The y-axis represents the average of correlations between centrality indices in the original sample and sample with case-dropped.

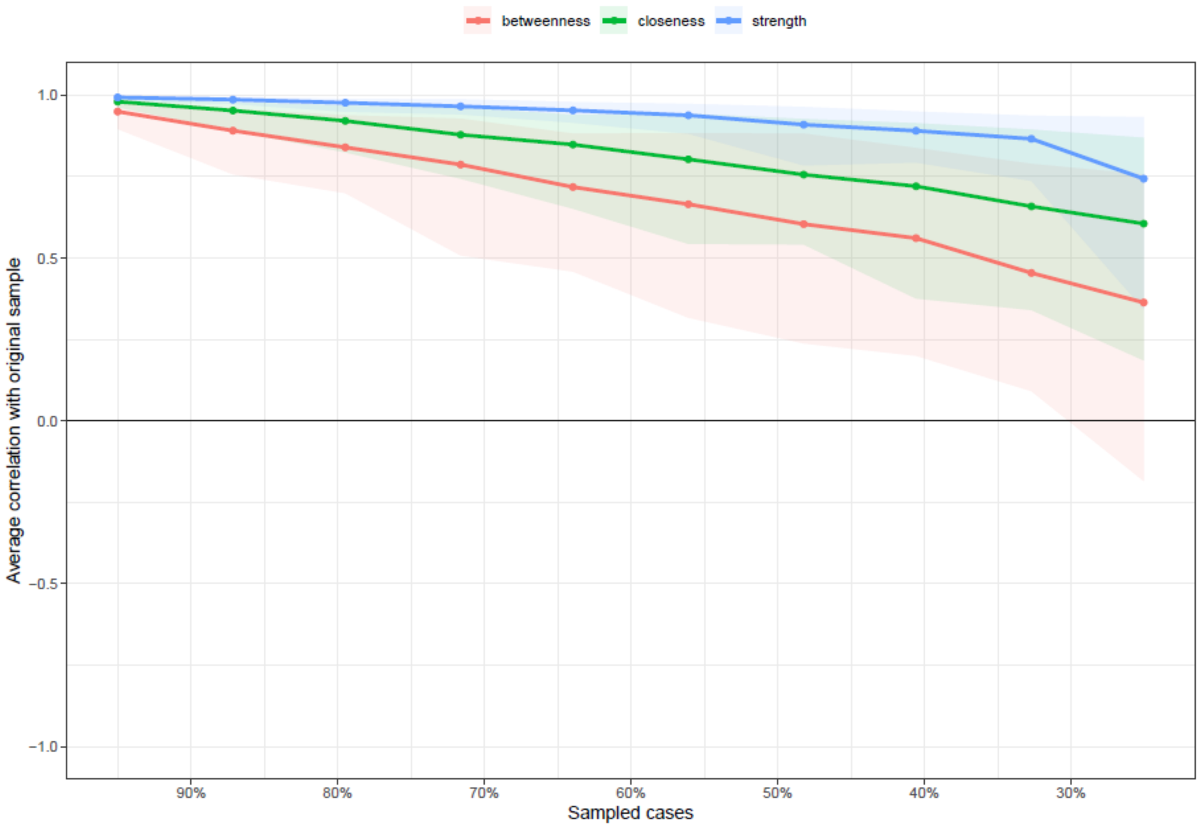

Figure S7. Symptom network structure of the Beck Anxiety Inventory in major depressive disorder patients with Zung Self-Rating Depression Scale scores ≥ 50 (n = 233). Node colors represent symptom communities identified by exploratory graph analysis (EGA).

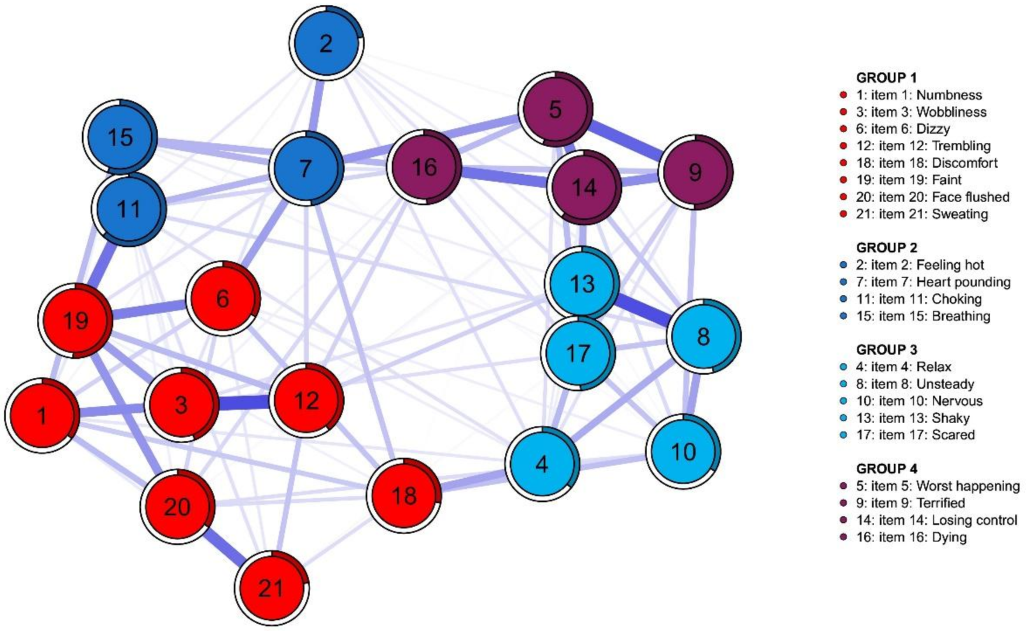

Figure S8. Symptom network structure of the Beck Anxiety Inventory in bipolar disorder patients with Zung Self-Rating Depression Scale scores ≥ 50 (n = 374). Node colors represent symptom communities identified by exploratory graph analysis (EGA).

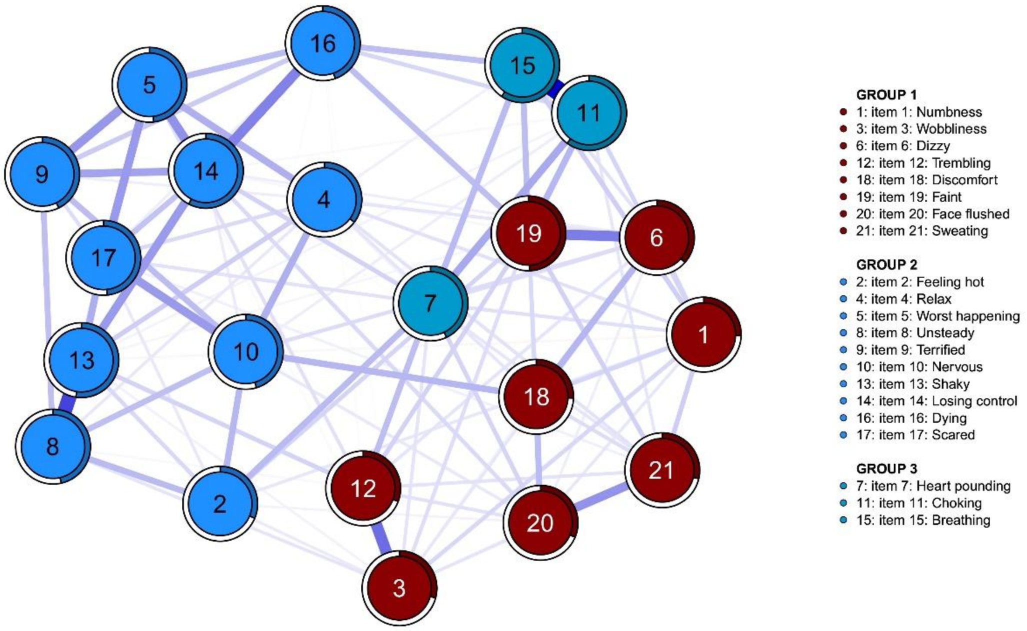

Figure S9. Bootstrapped difference tests between nodes in the BAI 21-item symptom network among major depressive disorder patients with Zung Self-Rating Depression Scale scores ≥ 50 (n = 233).

Note: Gray boxes indicate that the nodes are not significantly different from each other, while black boxes indicate that the nodes are significantly different (α = 0.05) from each other.

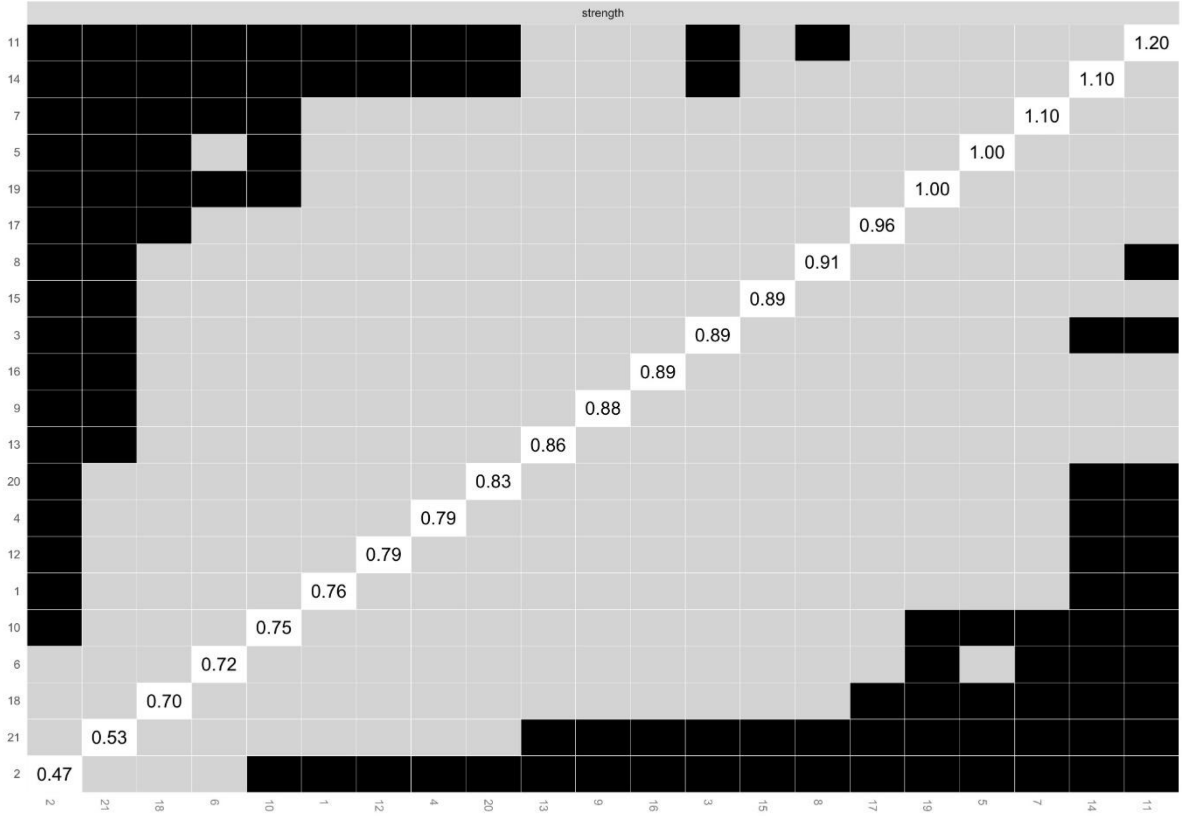

Figure S10. Bootstrapped difference tests between nodes in the BAI 21-item symptom network among bipolar disorder patients with Zung Self-Rating Depression Scale scores ≥ 50 (n = 374).

Note: Gray boxes indicate that the nodes are not significantly different from each other, while black boxes indicate that the nodes are significantly different (α = 0.05) from each other.

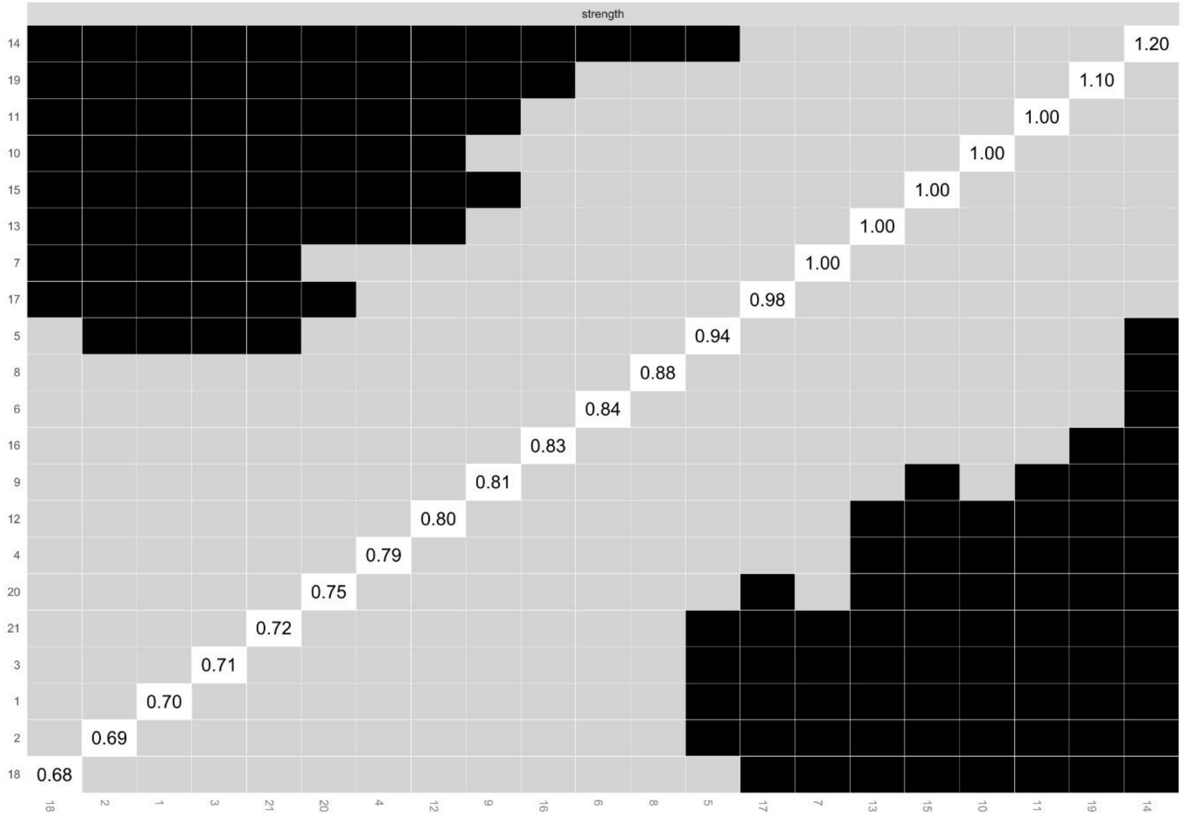

Supplement: Supplementary file 1 [file medicina-61-02245-s001.zip › medicina-4033794-supplementary.pdf]
